# Activation of conventional protein kinase C (PKC) is critical in the generation of human neutrophil extracellular traps

**DOI:** 10.1186/1476-9255-10-12

**Published:** 2013-03-21

**Authors:** Robert D Gray, Christopher D Lucas, Annie MacKellar, Feng Li, Katia Hiersemenzel, Chris Haslett, Donald J Davidson, Adriano G Rossi

**Affiliations:** 1 MRC Centre for Inflammation Research, The Queen's Medical Research Institute, University of Edinburgh Medical School, 47 Little France Crescent, Edinburgh, Scotland, UK

## Abstract

**Background:**

Activation of NADPH oxidase is required for neutrophil extracellular trap (NET) formation. Protein kinase C (PKC) is an upstream mediator of NADPH oxidase activation and thus likely to have a role in NET formation.

**Methods:**

Pharmacological inhibitors were used to block PKC activity in neutrophils harvested from healthy donor blood.

**Results:**

Pan PKC inhibition with Ro-31-8220 (p<0.001), conventional PKC inhibition with Go 6976 (p<0.001) and specific PKCβ inhibition with LY333531 (p<0.01) blocked NET formation in response to PMA. Inhibition of novel and atypical PKC had no effect. LY333531 blocked NET induction by the diacylglycerol analogue OAG (conventional PKC activator) (p<0.001).

**Conclusions:**

Conventional PKCs have a prominent role in NET formation. Furthermore PKCβ is the major isoform implicated in NET formation.

## Introduction

Neutrophil granulocytes are key cells of the innate immune system with a primary function of killing invading microorganisms such as bacteria, fungi and parasites to prevent pathogenic spread and invasion [1,2]. Once identified, neutrophils phagocytose and destroy microbes inside the phagolysosome by localised disgorgement of granule contents and the generation of reactive oxygen species (ROS) [3]. Engulfment of the microorganism allows killing to take place in a confined area within the cell and not in the extracellular space. Neutrophils may also liberate granule contents and ROS into the surrounding extracellular space to destroy nearby foreign pathogens. Dysregulation of these processes may cause histotoxic damage surrounding host cells. More recently a further extracellular killing mechanism available to neutrophils has been described known as neutrophil extracellular trap (NET) formation [3,4]. NETs are formed by the mixing of cytoplasmic contents with nuclear histones and DNA to form a network which is propelled to the exterior of the cell. Microbes are caught in this mesh and killed by the neutrophil proteins and histones contained in the NETs. This process of NET formation leads to a form of cell death, NETosis, that has been characterised as being different from either apoptosis or necrosis [5].

NET formation is known to be stimulated by specific cytokines (e.g., interleukin 8 (IL-8)), bacterial products (e.g., lipopolysaccharide (LPS)) and importantly by clinically relevant pathogens such as *Shigella flexneri*, *Staphylococcus aureus*, *Salmonella thyphimurium*, *Streptococcus pneumoniae* and the fungus *Candida albicans*[6]. Stimulation and activation of neutrophils with the diacylglycerol (DAG) mimetic phorbol 12-myristate 13-acetate (PMA) also results in the production of NETs and has given important clues as to the possible mechanism involved in the formation of such structures. It is clear that NET formation following PMA stimulation is dependent on ROS production (via the NADPH oxidase system) and this is likely to follow the activation of protein kinase C (PKC) as well as other pathways such as raf-MEK-ERK [7].

The PKC isozyme family is comprised of conventional, novel and atypical isoforms [8]. There are at least four conventional isozymes: PKCα, PKCβI, PKCβII and PKCγ. The novel isozyme group has four subtypes: PKCδ, PKCε, PKCη and PKCθ. The third group, atypical isozymes, consists of PKCζ and PKCι [9]. PMA stimulates conventional (α, βI, βII, γ) and novel (δ, ε, η, θ) PKC by mimicking the activating ligand DAG [8]. PKC isoforms of all classifications have been reported in neutrophils from healthy donors [10]. Given that PMA activation triggers NET formation, we hypothesised that specific isoform(s) of PKC are a key modulator of the NET formation pathway. To address this hypothesis we evaluated a panel of PKC inhibitors on NET formation.

## Material and methods

### Reagents

Dihydrorhodamine (DHR), dimethyl sulfoxide (DMSO), diphenyliodonium (DPI), Phorbol 12-myristate 13-acetate (PMA), Ro-31-8220, PKCζ pseudosubstrate myristoyl trifluoroacetate (PKCζ inhibitor) and SYTOX green were purchased from Sigma-Aldrich (Dorset, UK); Rottlerin, Gö 6976 and LY333531 were from Calbiochem (Merck) (Darmstadt, Germany).

### Isolation of human neutrophils

Peripheral blood neutrophils were isolated from healthy human volunteers according to Lothian Research Ethics Committee approvals #08/S1103/38 via dextran sedimentation and Percoll™ discontinuous gradients as described [11,12]. Informed written consent was obtained from all subjects. Purity of the neutrophils was assessed by examination of cytocentrifuge preparations and was greater than 95%.

### Assessment of NET formation

Neutrophils (5×10^4^ cells/well) in HBSS containing Ca^2+^, Mg^2+^ and Hepes (20 mM) were aliquoted (180 μl) into 96 well plates and left to settle for 30 min at 37°C. The inhibitors Ro-31-8220, DPI, rottlerin, PKCζ inhibitor, Gö 6976 and PKCβ inhibitor were added at appropriate concentrations to wells in duplicates and incubated for 30 min before adding PMA. The final volume in each well was 200 μl. Plates were incubated for 4 h and then SYTOX green (6 μM final concentration), a cell-impermeable nucleic acid stain, with an excitation/emission maxima of 504/523 nm to give a green fluorescent light, was added and NET formation was observed by measuring mean fluorescence in 96 well plates. In some experiments 1-oleoyl-2-acetyl-sn-glycerol (OAG) was used to stimulate cells in place of PMA. Results were evaluated by measuring the mean fluorescence in 96 well plates after the subtraction of background fluorescence. Cells were also visualised by fluorescent microscopy carried out on a Zeiss Axiovert S100 fluorescent microscope (Carl Zeiss, Germany) and an Evos fl inverted microscope (AMG, Bothwell, WA).

### Statistical analysis

Data were assessed by one way ANOVA followed by a post-hoc Dunnett’s test. The data were expressed as mean ± standard error of the mean (SEM), and values of p < 0.05 were considered statistically significant. All statistics were performed using GraphPad Prism 5 software (GraphPad, CA, USA).

## Results

### PMA induced NET formation

**Figure 1:**
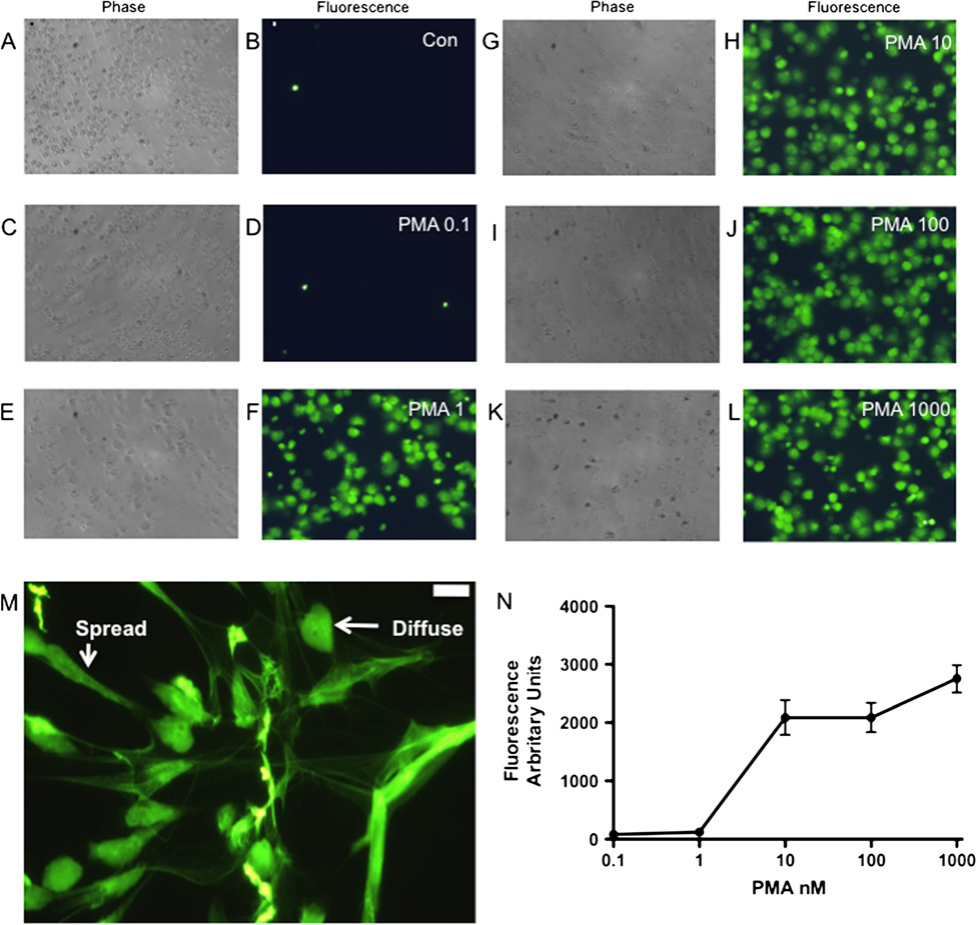
**PMA induced NET formation: A-M) Cells were treated for 4 h with PMA (A,B: control, C,D: 0.1 nM, E,F 1 nM, G,H 10 nM, I,J 100 nM, K,L 1 μM,) and microscopy performed at a density of 50,000 cells per well in a 96 well plate.** Sytox Green cell impermeable dye was added at a concentration of 6 μM. Cells with a typical morphology of diffuse and spread NETs were noted [in this figure 50,000 cells were plated in 1 ml of media in plastic 24 well plates and stimulated as above with 100 nM PMA] (**M**). Scale bars = 10 microns. A greater abundance of NETs was seen with increases in PMA concentration but abundance was similar in the 10–1000 nM range. **N**) Fluorescence of extracellular DNA by cell impermeable Sytox Green was measured with an excitation of 485 nm and measured at 530 nm. Background fluorescence was subtracted and the abundance of NETS expressed as fluorescence in arbitrary units. The concentration-dependent response of NET formation plateaued after 10 nM PMA. Data show mean +/− SEM for n = 6 independent experiments.

Incubation of human neutrophils with PMA induced dramatic changes in morphology at 4 h after stimulation, resulting in NETs that stained positive with SYTOX green which is impermeable to cells with an intact membrane. The abundance of NETs was almost maximal at 10 nM PMA above which the magnitude of NET formation plateaued (Figure 1A-N). Cells stimulated with PMA demonstrated typical morphology of diffuse and spread NETs (Figure 1M). Measuring the level of total fluorescence with SYTOX green allowed the assessment of total extracellular DNA and thus NET formation (Figure 1N). NET formation determined by microscopy and cell counting (i.e., by expressing the number of areas of extracellular DNA as a percentage of total cell count [14]) strongly correlated with the measurement of NET abundance based on total fluorescence (r^2^=0.98), data not shown. Therefore total fluorescence was utilised as a reliable screening assay in further experiments to allow a range of inhibitors to be compared, before confirmation with gold-standard microscopic validation. This test was reproducible with an average inter-assay coefficient of variation of 14.3%. As almost maximal NET formation was gained with 10 nM PMA (Figure 1B), this concentration was selected for all further experiments.

### NET formation is PKC and NADPH oxidase dependent

**Figure 2:**
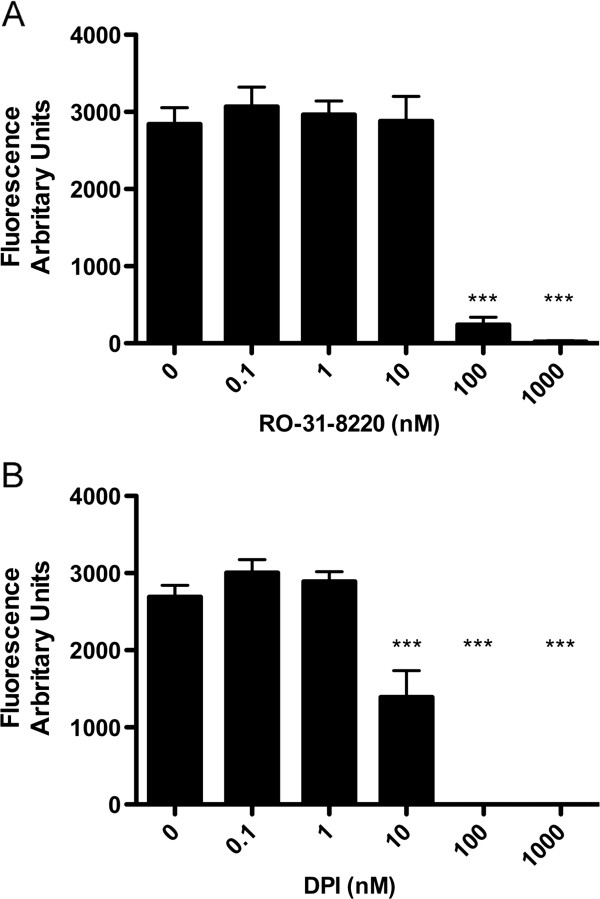
**Effect of DPI and Ro-31-8220 on NET formation: Cells were plated as described and either pre-treated with DPI or Ro-31-8220 at increasing concentrations, or control media, before stimulating with PMA at 10 nM for 4 h.** NET abundance was measured by total fluorescence from Sytox Green cell impermeable dye. **A**) Pre-treatment with Ro-31-8220 completely abrogated NET formation at 100 nM and 1 μM. Data show mean +/− SEM for n=4 independent experiments. *** indicates p<0.001. **B**) Pre-treatment with DPI significantly decreased NET formation at 10 nM, and completely abrogated NET formation at 100 nM and 1 μM. Data show mean +/− SEM for n=4 independent experiments. *** indicates p<0.001.

In order to determine whether NET formation was dependent on the activation of PKC and NADPH oxidase, cells were preincubated with increasing concentrations of the specific but isozyme non-selective PKC inhibitor, Ro-31-8220 or the NADPH inhibitor diphenyloidonium (DPI) for 30 min and then treated with 10 nM PMA. Both Ro-31-8220 and DPI, completely inhibited PMA induced NET formation (Figure 2A and 2B) measured by SYTOX green fluorescence and confirmed by microscopy. These data confirm the key role of NADPH oxidase and demonstrate that the NET-forming activity of PMA is critically dependent upon PKC pathways upstream of NADPH oxidase.

### Specific PKC isoforms regulated NET formation

**Figure 3:**
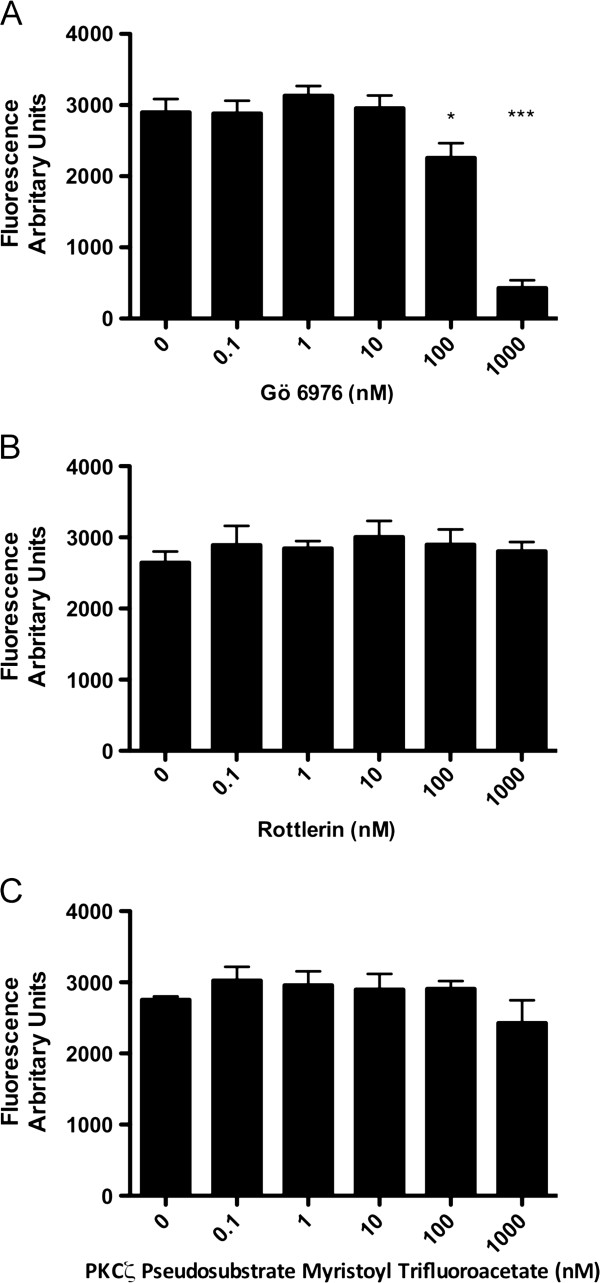
**Effect of isozyme specific PKC inhibitors on NET formation: Cells were plated as described and either pre-treated with at increasing concentrations of Gö 6976 (conventional PKC inhibitor), Rottlerin (atypical PKC inhibitor), PKCζ psuedosubstrate (novel PKC inhibitor), or control media, before stimulation with PMA at 10 nM for 4 h. A**) Pre-treatment with Gö 6976 significantly reduced NET formation at 100 nM (P<0.05) and 1 μM (p<0.001). **B**) Pre-treatment with Rottlerin had no effect on NET formation. **C**) Pre-treatment with PKCζ psuedosubstrate had no effect on NET formation. Data show mean +/− SEM for n=3 independent experiments. * indicates p<0.05, *** indicates p<0.001.

Ro-31-8220 is an inhibitor of multiple PKC isoforms, therefore in order to investigate PKC isoform specificity, PKC isoform classes were targeted with specific inhibitors; Gö 6976 (Conventional), rottlerin (Novel), PKCζ psuedosubstrate (Atypical) (Figure 3A, B, C). Figure 3A demonstrates that Gö 6976 significantly inhibited NET formation at 100 nM (p<0.05) and 1 μM (p<0.001). In contrast, Rottlerin and PKCζ psuedosubstrate had no significant effect on NET formation (Figure 3B and C). These data were confirmed microscopically and demonstrate a key role for conventional PKC in NET formation.

### PKC β is Primarily Implicated in NET formation in Response to PMA and OAG

**Figure 4:**
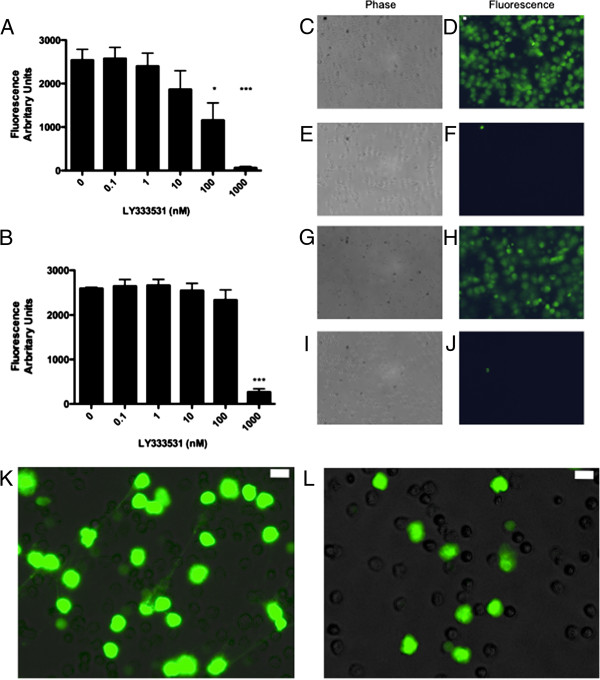
**Effect of PKC β on NET formation in response to PMA and DAG analogue OAG: Cells were plated as described and pre-treated with increasing concentrations of LY333531 (PKCβ inhibitor) before treatment with either 10 nM PMA or 20 μM 1-oleoyl-2-acetyl-sn-glycerol (OAG) for 4 h. A**) Pre-Treatment with increasing concentrations of LY333531 decreases NET formation in response to 10 nM PMA. Data show mean +/− SEM for n=6 independent experiments for PMA stimulated cells and n=3 for OAG stimulated cells. *indicates p<0.05, ***p<0.001. **B**). Pre-treatment with increasing concentrations of LY333531 completely abrogated NET formation in response to 20 μM OAG. Data show mean +/− SEM for n=3 independent experiments. *** indicates p<0.001. **C**-**J**). Pre-treatment with 1 μM LY333531 completely abrogated NET formation in response to PMA and OAG, images representative of n=3 independent experiments (**C**,**D** Cells treated with 10 nM PMA. **E**,**F** cells pre-treated with 1 μM LY333531 for 30 min then 10 nM PMA. **G**,**H** cells treated with 20 μM OAG. **I**,**G** Cells pre-treated with 1 μM LY333531 for 30 min then 20 μM OAG. **K**,**L** Higher magnification images of effects of treatment with 100 nM LY333531 (**L**) prior to 10 nM PMA. Fluorescent and phase images combined. There is an absence of spreading NET material in the LY333531 treated cells although some diffuse NET formation is still present at this concentration of inhibitor. Scale bars = 10 microns.

Gö 6976 is an inhibitor of both PKCα and β, thus, in order to separate the roles of these two isozymes, a specific PKCβ inhibitor (LY333531) was employed (Figure 4). LY333531 significantly inhibited NET formation by PMA at a concentration of 100 nM (p<0.05) and 1 μM (p<0.01) (Figure 4A). In addition, 1-oleoyl-2-acetyl-sn-glycerol (OAG; a DAG analogue and activator of conventional PKC isoforms) stimulated NET formation in a manner similar to that of PMA (albeit at higher concentrations) and was also significantly inhibited by 1 μM LY333531 (p<0.01) (Figure 4B). These data were confirmed by microscopy and delineate a central role for PKC β in PMA and OAG-induced NET formation (Figure 4C-J). Interestingly as well as knocking down total NET production LY333531 completely abrogated the presence of “spreading NETs” at the 100 nm concentration but some diffuse NET formation was still observed (Figure 4K,L).

### PKCβ inhibition has downstream effects on oxidative burst

**Figure 5:**
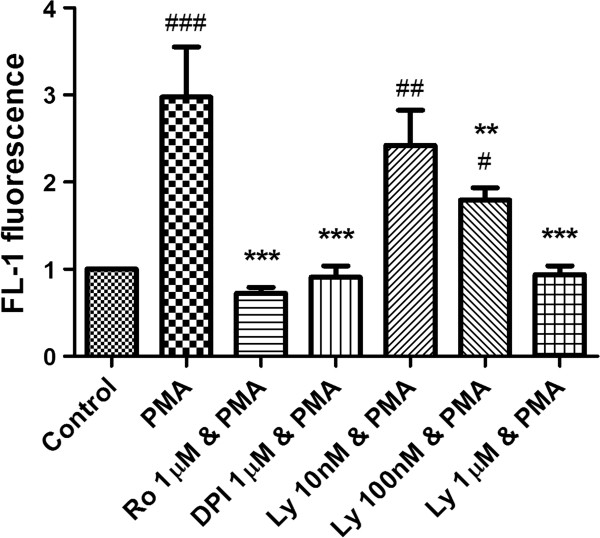
**The downstream effect of PKC β inhibition on oxidative burst.** Cells were pretreated with LY333531 (Ly) at increasing concentrations and NADPH oxidase activity measured by DHR fluorescence on flow-cytometry. DPI and Ro-31-8220 (Ro) were used as positive controls. LY333531 reduced NADPH oxidase activity at both 100 nM (p<0.01) and 1 μM (p<0.001) consistent with the concentrations of inhibitor required to reduce NET formation. # indicates p<0.05, ## indicates p<0.01, and ### indicates p<0.001 vs. untreated control. ** indicates p<0.01 and *** indicates p<0.001 vs. PMA treated cells.

To elucidate the downstream effects of PKCβ inhibition we assessed the effects of LY333531 on oxidative burst as measured by DHR fluorescence on flow cytometry. LY333531 reduced NADPH oxidative burst at the same concentrations required to reduce NET formation, with a partial but significant knock down of activity at 100 nM (p<0.01) and a complete knockdown at 1 μM (p<0.001) in comparison to the positive controls of DPI and Ro-31-8220 (Figure 5).

### Assessment of downstream effects of PKCβ inhibition with specific inhibitors

ROS generation was assessed by dihydrorhodamine (DHR) fluorescence as described previously [13]. Neutrophils were resuspended in HBSS with cations and loaded with DHR (2 M; Invitrogen, Carlsbad, CA, USA) for 10 min. Cells were then incubated with or without PKCβ inhibitor (at 10, 100 and 1000 nM) or the positive controls Ro-31-8220 or DPI at 1μM on a shaking heat block for 30 min before stimulation with PMA 10 nM for a further 15 min. DHR fluorescence was analyzed by flow cytometry (FL-1).

## Discussion

The results clearly show that NET formation induced by PMA is PKC and NADPH oxidase dependent. NET formation was blocked by both Pan-PKC inhibition and conventional-PKC inhibition. Furthermore, a specific PKCβ inhibitor (LY333531) also blocked NET formation. LY333531 has high selectivity for PKCβ over other conventional isoforms (IC50 of around 5 nM) with a 60 fold selectivity for PKCβ over PKCα [15]. At higher concentrations specific inhibitors may have non-selective effects on other PKC isoforms. The IC50 of LY333531 for PKCα is around 300 nM, suggesting that the majority of the effect of this compound at the concentrations utilised in our study is via the inhibition of PKCβ and not PKCα; this is evidenced by the significant reduction in NET formation with 100 nM LY333531. The intracellular concentration of LY333531 within the neutrophil following incubation is unknown but it is unlikely to be fully absorbed and as such again we would suggest the effects are due to inhibition of PKCβ. Previous work has demonstrated that PKCβ accounts for 50% of the neutrophil response to PMA further underlining the likely predominant role of PKCβ in NET production [16].

Oxidative burst and the generation of reactive oxygen species including superoxide anions (0_2_^-^) and nitric oxide (NO) are fundamental responses of the neutrophil to inflammatory stimuli and pathogens. NET formation is dependent on NADPH oxidase activation and consequently on the generation of 0_2_^-^ which can be blocked by DPI. DPI inhibits NADPH oxidase by binding to specific subunits in the enzyme complex and preventing electron flow and 0_2_^-^ production [17]. The main component of NADPH oxidase is the flavocytochrome b558, a dimer of p22phox and gp91phox, which is an active transporter of electrons across the membrane. Coupled to these are proteins p40phox, p47phox, p67phox and p21rac which are crucial to electron translocation [18]. These proteins assemble when activated to produce 0_2_^-^ which are then spontaneously converted to H_2_O_2_. Interestingly, p47phox has to be phosphorylated to acquire a conformational rearrangement to expose the domains that are important for the NADPH oxidase function, and this phosphorylation is mediated by PKC [19]. This is consistent with our findings that PKC is involved in PMA induced NET formation and furthermore that PKCβ is the isoform crucially involved. This is further underlined by the finding that oxidative burst is reduced by concentrations of LY333531 that reduce NET formation.

The beneficial anti-microbial effects of NET formation have been described in several studies [4,20-25]. Indeed, this is perhaps most pertinently displayed in restoration of NADPH oxidase function in chronic granulomatous disease by gene therapy leading to an increased resistance to fungal infection and clinical improvement secondary to the restoration of the ability to form NETs [22]. Several studies however have demonstrated a pro-inflammatory potential of NETs in a diverse range of diseases including systemic lupus erythematosus [26-28], cystic fibrosis [1,29,30] and psoriasis [31]. Therefore the modulation of NET production may be a viable anti-inflammatory target. Inhibition of PKC activity represents one such target as PKC inhibitors have been in development for many years as potential anti-cancer therapies, many of which are orally bioavailable [9]. Furthermore the relative redundancy in PKC function due to multiple isoforms may allow the targeting of specific PKCs in specific cell types at specific organ sites. PKCβ knock out in a murine model has been demonstrated to modulate ischemia reperfusion injury *in vivo*[32], however these mice may also be immunodeficient [33] and thus caution must be exercised in any strategy to specifically target PKC. Extracellular traps from both neutrophils and mast cells have been demonstrated in psoriatic skin lesions and from purified neutrophils from psoriasis patients in association with IL-17 and MPO, directly implicating extracellular traps in the pathogenesis of disease [31]. A previous study of a PKC inhibitor AEB071 with specificity for PKC α, β, and θ in psoriasis demonstrated not only *in-vitro* effects on T cell proliferation and cytokine production but also a clinical improvement in psoriatic lesions in treated patients [34]. We may infer that some of this effect may be due to a direct effect of PKC inhibition on NET formation and thus inflammation in the skin lesions of these patients. Further studies will of course be required to support this hypothesis.

In summary, NET formation in response to PMA and DAG analogues is dependent on PKC activation. Furthermore, we demonstrate that conventional PKC and in particular PKCβ is the predominant isoform responsible for NET formation under these conditions. Although NETs have been demonstrated to entrap and kill various microorganisms there is burgeoning evidence implicating a role for these structures in inflammatory disease and potential modulation of NET production (by PKC inhibition) may offer a novel anti-inflammatory strategy.

## Competing interests

The authors declare that they have no competing interests.

## Authors’ contributions

RDG, CDL, AM, FL and KM carried out the experiments. RDG, CH, DJD and AGR designed the experiments and provided a critical review of methods. RDG drafted the manuscript. All authors read and approved the final manuscript.

## Funding

RDG is a Wellcome Trust Fellow (093767). DD is an MRC Senior Research Fellow (G1002046). This work was also funded by the Wellcome Trust (WT094415; CL) and the MRC (G0601481; AGR and CH).
